# The Association Between Major Depression and Alzheimer’s Disease Risk: Evidence from a 12-Year Longitudinal Study

**DOI:** 10.3390/jcm13237039

**Published:** 2024-11-21

**Authors:** Anais Sevil-Pérez, Raúl López-Antón, Patricia Gracia-García, Concepción de la Cámara, Ana Gascón-Catalán, Javier Santabárbara

**Affiliations:** 1 Department of Physiatry and Nursing, University of Zaragoza, 50009 Zaragoza, Spain; anais93_1@hotmail.com (A.S.-P.); agascon@unizar.es (A.G.-C.); 2 Faculty of Health Sciences, Universidad San Jorge, 50830 Zaragoza, Spain; 3 Department of Psychology and Sociology, University of Zaragoza, 50009 Zaragoza, Spain; 4 Centro de Investigación Biomédica en Red de Salud Mental (CIBERSAM), Ministry of Science and Innovation, 28029 Madrid, Spain; pgraciagarcia@gmail.com (P.G.-G.); conchidlc@hotmail.com (C.d.l.C.); jsantabarbara@unizar.es (J.S.); 5 Department of Medicine, Psychiatry and Dermatology, University of Zaragoza, 50009 Zaragoza, Spain; 6 Instituto de Investigación Sanitaria Aragón (IIS Aragón), 50009 Zaragoza, Spain; 7 Psychiatry Service, Hospital Universitario Miguel Servet, 50009 Zaragoza, Spain; 8 Psychiatry Service, Hospital Universitario Lozano Blesa, 50009 Zaragoza, Spain; 9 Department of Microbiology, Pediatrics, Radiology and Public Health, University of Zaragoza, 50009 Zaragoza, Spain

**Keywords:** depression, dementia, Alzheimer’s disease, longitudinal study

## Abstract

**Background:** The relationship between depression, particularly major depression (MD), as a risk factor for Alzheimer’s disease (AD) is well established; however, its precise role remains contested. Findings from the fourth wave of the ZARADEMP longitudinal study provide further insights into the association between MD and AD risk. **Objectives**: This study aimed to examine the association between MD and incident AD, controlling for established risk factors. **Methods**: The study analyzed 4803 participants, of whom 4057 were followed over a 12-year period as part of the ZARADEMP longitudinal study. Depression was assessed using the GMS-AGECAT, and dementia was diagnosed according to DSM-IV criteria. The association between MD and incident AD was evaluated using Cox proportional hazards regression models. **Results**: The incidence of AD was approximately twice as high in participants with MD compared to those without (relative risk = 2.07; 95% CI: 0.85–5.03; *p* = 0.123). This risk was nearly threefold higher in the fully adjusted model. **Conclusions**: These findings underscore a significant association between MD and an increased risk of AD, emphasizing the need for vigilant monitoring and potential early intervention among individuals diagnosed with MD.

## 1. Introduction

Geriatric depression, defined as depression emerging in individuals over 65 years without a prior history of depressive episodes, is marked by persistent sadness often linked to life challenges unique to aging, such as economic hardship, physical disability, social isolation, relocation, caregiving, and bereavement. These experiences contribute to considerable suffering, family strain, and functional impairment [1,2,3]. Depression prevalence in older adults is notably high, underscoring the critical need for preventive and therapeutic interventions, particularly given its associations with frailty and elevated suicide risk within this demographic [4].

Depression has been proposed as a potential risk factor for dementia. However, some authors argue that it may serve merely as a prodromal phase of dementia rather than an independent risk factor [5,6,7,8]. A meta-analysis conducted by our research group investigated the association between depression and dementia risk, finding a significant link with a pooled relative risk of 1.63 and a population-attributable fraction of 8.6% for dementia among individuals with depression [9]. In a separate meta-analysis focusing on clinically significant depression, we identified an increased risk for both dementia and Alzheimer’s disease (AD), with a population-attributable fraction of 10.8% for AD [10]. These findings underscore the potential benefit of early intervention in depressive disorders to mitigate dementia risk.

Longitudinal studies remain the gold standard for this area of research as they enable patient tracking over extended periods, facilitating causal inferences about enteropathogenic mechanisms and informing preventative strategies aimed at reducing the incidence of both conditions [11]. For instance, data from the ZARADEMP study, a longitudinal, three-wave epidemiologic investigation, followed a cohort of individuals aged 55 years and older, with assessments at 2.5- and 4.5-year intervals. This study reported that severe depression heightened the risk of AD, even after accounting for the competing risk of mortality. However, the relatively short follow-up duration limits the generalizability of these findings [12]. Furthermore, no longitudinal studies have yet explored this relationship over follow-up periods exceeding 10 years, leaving a gap in understanding the long-term effects of depression on AD risk.

Despite extensive research, critical gaps persist regarding the influence of specific depression subtypes—particularly major depression—on AD risk. Major depression, a severe form of depression, may involve unique neurobiological mechanisms that specifically elevate AD risk, a hypothesis insufficiently examined in prior studies [13].

Against this context, this study aims to elucidate the risk of AD in patients diagnosed with major depression while controlling for established known risk factors [14]. Understanding the association between major depression and AD is crucial for clinical practice, as it could guide the development of targeted screening and intervention strategies. Such insights could ultimately contribute to delaying or preventing AD onset among high-risk individuals with major depression, enhancing their quality of life and long-term outcomes [15].

## 2. Materials and Methods

### 2.1. Study Design and Population

The sample for the present study was drawn from the ZARAgoza DEMencia y DEPresión (ZARADEMP) project [16], a longitudinal epidemiological study conducted in four waves in Zaragoza, Spain. This study was designed to investigate the incidence and risk factors of dementia and its major subtypes, as well as depression, in an adult population aged 55 years and older. For this purpose, a random sample of community-dwelling individuals was drawn from the official 1991 census lists, stratified with proportional allocation by age and sex. Detailed baseline sociodemographic and clinical characteristics have been previously documented [16,17,18]. The sample size was calculated to study the risk factors for incident dementia, the main objective of the project, considering dropout information from a previous study [19]. In the cross-sectional study, the refusal rate was 20.5%, and 4803 individuals were interviewed at the beginning of the study (Wave I, 1994). Strict criteria were applied to include only cognitively healthy individuals in the follow-up cohort. Individuals with any type of dementia or cognitive impairment at the beginning of the study, according to the Geriatric Mental State-Automated Geriatric Examination for Computer Assisted Taxonomy package criteria [20], were excluded from the follow-up assessments (Wave II, 1997; Wave III, 1999; Wave IV, 2006), leaving a sample of 4057 for this study.

The procedure of testing has been previously published elsewhere [16,18]. In brief, the diagnostic and detection process was carried out in two stages. In the first stage, trained interviewers, regularly supervised, conducted the ZARADEMP interview in the homes or residences of the participants. The ZARADEMP interview, designed for this study, incorporates the validated Spanish version of multiple international instruments: the Mini-Mental State Examination (MMSE) [21], the Geriatric Mental State (GMS-AGECAT) [20], the History and Aetiology Schedule (HAS) [22], the Lawton and Brody scale [23], the Katz Index [24], and the EURODEM questionnaire [25]. In the second stage, psychiatrists trained for this research and who supervised the interviewers re-evaluated all those individuals who were considered “possible psychiatric cases” after the first stage, as well as those individuals whose available information was deemed unreliable. These interviews were also conducted in the participants’ homes and used the same instruments as in the first phase, as well as the Hachinski scale [26] and a brief standardized neurological examination. The final diagnosis of dementia (and subtype) following DSM-IV criteria [27] was made by consensus, requiring agreement from at least three psychiatrists from a four-member panel. To document the accuracy of this panel, a percentage of the detected cases were invited to participate in a diagnostic round at the hospital, where neuroimaging studies and neuropsychological evaluations were conducted, achieving high diagnostic agreement (95.8% for dementia cases and 87.5% for dementia subtype cases).

### 2.2. Assessment and Diagnosis of Depression

The diagnosis of depression was based on the GMS-AGECAT stepped approach. This method is valid for detecting “depression requiring clinical attention” in community samples. Psychotic depression, classified under the GMS-AGECAT system, is considered analogous to major depression (MD). This classification follows the diagnostic framework established by Copeland et al. [20], which defines psychotic depression as equivalent to major depression, while neurotic depression aligns with minor depression. Previous studies in this sample [12] have demonstrated that these subtypes carry distinct risks for neurocognitive outcomes, particularly Alzheimer’s disease. In this study, ‘MD’ refers to major depression, as defined by the DSM-IV criteria. Following the original GMS-AGECAT protocol, after symptom evaluation (Stage I), a diagnosis of depression emerged in Stage II. In this stage, a computer program (the Automatic Geriatric Examination for Computer Assissted Taxonomy (AGECAT)) compares syndrome clusters (e.g., dementia, depression, anxiety) to reach a final diagnosis, recorded as “subsyndromal” (confidence levels 1 and 2) or as a diagnostic “case” (confidence levels ≥3). AGECAT “caseness” implies the “desirability of intervention”. Additionally, “cases” of depression are classified as “severe depression” (including melancholic symptoms) and “non-severe depression”. Subsyndromal in this system implies that the psychopathological symptoms are not severe enough to warrant intervention. Information from the History and Aetiology Schedule was used to define the onset of depression and the use of antidepressant treatment (classified according to the Anatomical Therapeutic Chemical Classification system codes N06A* and/or N06C1A). Depression was defined as persistent if it was present at baseline and the first follow-up assessment (Wave II). For this study, 729 participants were diagnosed with depression (113 diagnosed with major depression) or depressive symptoms at baseline using the GMS-AGECAT criteria. This subgroup was followed longitudinally to assess the progression of depressive symptoms and their association with the development of Alzheimer’s disease.

### 2.3. Covariates

The potential confounding factors evaluated at the beginning of the study included sociodemographic characteristics (age, sex, educational level, living arrangement, marital status, employment status, and social class) and clinical risk factors, which included cardiovascular risk factors (hypertension, diabetes, cardiac issues, vascular issues, and number of cardiovascular events) and cognitive status (MMSE score).

The choice of covariates, including age, sex, educational level, and cardiovascular risk factors, is based on extensive research identifying these factors as strong predictors of Alzheimer’s disease and depression in older adults [12,28].

Education was initially classified into three levels: illiterate (unable to read and write, and less than 2 years of schooling), primary (complete or incomplete), and secondary or higher. Blood pressure was measured based on the average of two readings during the interview using a standard manual sphygmomanometer; hypertension was defined as blood pressure > 140/99 mmHg or if the participant reported receiving treatment for hypertension. The presence of vascular risk factors, cardiac issues, and diabetes was determined from the medical history obtained using the EURODEM Risk Factors Questionnaire [25]. Vascular and cardiac issues were dichotomized, distinguishing between prior vascular or cardiac disease (angina pectoris and/or myocardial infarction and/or stroke) and no history of vascular or cardiac disease. Diabetes was dichotomized into those with a prior medical diagnosis or receiving treatment for diabetes and those without diabetes. A new variable was created to group cardiovascular risk factors as a sum of the pathologies included in this category.

### 2.4. Data Analysis

A descriptive analysis was conducted to outline the characteristics of each study variable. For the sociodemographic table, chi-square tests and *t*-tests were applied to examine categorical and continuous variables, respectively.

To investigate the association between major depression (MD) and Alzheimer’s disease (AD) risk, we employed a multivariate survival analysis using Cox proportional hazards regression, with years of follow-up as the time-scale variable. An intention-to-treat approach was applied, excluding participants who had moved away, were deceased, refused participation, or were untraceable during follow-up [29]. Initially, we conducted an unadjusted analysis to establish a baseline hazard ratio for MD and AD risk. In a stepwise approach, covariates were sequentially incorporated into the model to assess their influence on the association. In the first step, each variable was individually added to the baseline model. In the second step, variables grouped within the same category were entered as a block. In the final step, all covariates were simultaneously entered into the model. For variables that did not meet statistical significance (*p* < 0.05), an additional analysis was conducted exclusively with those that reached statistical significance, ensuring robust effect estimation.

All analyses were conducted using IBM SPSS Statistics, version 26.

### 2.5. Ethical Considerations

The study was approved by the Research Ethics Committees of the University of Zaragoza and the Hospital Clínico de Zaragoza, in compliance with Spanish law. Throughout the study, the principles of the Helsinki Declaration [30] regarding written informed consent, confidentiality, and privacy were strictly adhered to.

## 3. Results

**Figure 1:**
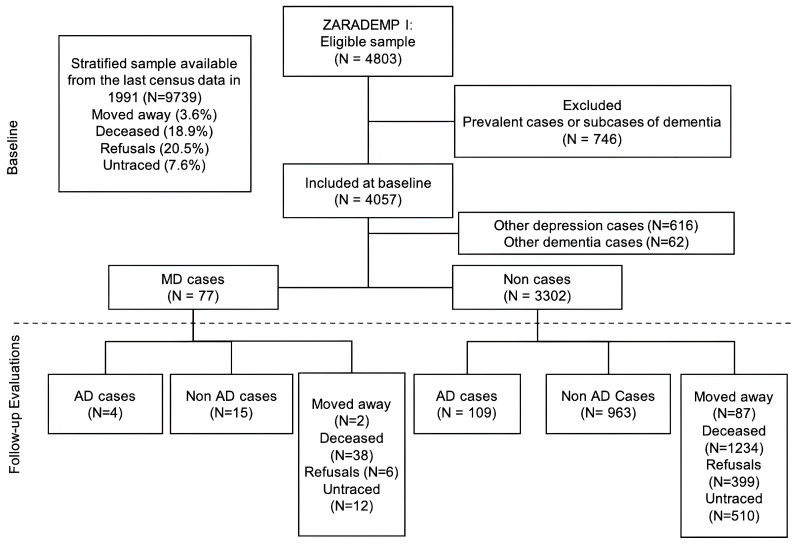
Flowchart of study.

Of the 4803 individuals initially interviewed, 746 exhibited signs or symptoms of dementia or cognitive impairment at baseline (Wave I) and were therefore excluded from longitudinal follow-up. Among the remaining 4057 participants included at baseline, 678 were excluded for having other forms of depression or other dementia subtypes. Among the participants included in the follow-up period, 77 were classified as major depression (MD) cases and 3302 were non-cases (Figure 1).

We obtained data on the incidence of Alzheimer’s disease (AD) among both MD and non-MD participants (Figure 1). During follow-up, 4 participants in the MD group developed incident AD, while 15 did not. Additionally, there were further losses among MD cases due to participants moving away (*n* = 2, 2.6%), deaths (*n* = 38, 49.4%), refusals (*n* = 6, 7.8%), and untraceable cases (*n* = 12, 15.6%). In the non-MD group, 109 participants developed AD, while 963 remained non-AD and non-MD cases This group also experienced losses due people moving away (*n* = 87, 2.2%), deaths (*n* = 1234, 31.0%), refusals (*n* = 399, 10.0%), and untraceable cases (*n* = 510, 12.8%). Percentages are calculated as the proportion of each type of loss within each group based on the respective group totals.

In the study sample, 2229 were women (54.9%). The mean age was 72.08 years (±9.16). Regarding educational level, 315 (7.8%) were “illiterate”, 3019 (75%) had primary education, and 690 (17.1%) had secondary or higher education, with a total of 33 cases lost (0.8%). Regarding biomedical variables, there were more participants with hypertension (2457; 67.8%) and one or more cardiovascular diseases (2329; 60.2%) than with other conditions, and 93 (2.3%) individuals had low MMSE scores.

Table 1 presents the baseline sociodemographic and clinical characteristics of the study participants, comparing those with major depression (MD) and those without MD (non-cases). Participants with MD were slightly younger compared to non-cases, though this difference was not statistically significant. A significant sex difference was observed, with a statistically significant higher proportion of females in the MD group compared to the non-MD group (*p* < 0.001). Educational level also showed significant variation between groups: a lower percentage of participants with MD had secondary education or higher compared to non-cases (*p* < 0.05). Regarding clinical factors, no significant differences were found between the MD and non-MD groups in terms of diabetes or hypertension. However, the incidence of previous heart disease and previous vascular disease was slightly higher in the MD group, though these differences did not reach statistical significance. The number of cardiovascular illnesses exhibited a significant difference, with fewer participants in the MD group reporting no cardiovascular illness compared to non-cases (*p* < 0.001). Additionally, differences in cognitive function, as measured by the Mini-Mental State Examination (MMSE), were substantial, with the MD group showing a markedly lower mean MMSE score than the non-cases group (*p* < 0.001).

The proportion of incident cases of AD was associated with the severity of the depression status: 10.17% for non-cases and 21.05% for MD cases (relative risk = 2.07; 95% CI: 0.85–5.03; *p* = 0.123) (Figure 1).

Table 2 shows the results of the Cox regression analysis of AD incidence associated with major depression status at baseline. In the multivariate analysis, we found an almost 3-times higher Alzheimer’s disease risk in participants with major depression, adjusted for age, gender, and educational level, compared with non-cases (*p* = 0.044). This magnitude of effect remains when cardiovascular risk factors, sociodemographic factors, and MMSE score are controlled (HR = 2.81; *p* = 0.053).

## 4. Discussion

Our results support the hypothesis that major depression (MD) increases the risk of developing Alzheimer’s disease (AD). This finding reinforces previous studies suggesting that depression may act not only as a risk factor but also as a clinical precursor of AD, especially in older adults [9,12]. The high incidence rate of AD observed in individuals with MD (HR: 2.81; 95% CI: 0.98–8.01) highlights the need for careful monitoring of patients with severe psychiatric symptoms, as they may be at increased risk of long-term cognitive impairment.

Our results also suggest that patients with MD are a particularly vulnerable subgroup. The distinction between MD and other forms of depression is relevant for understanding the possible heterogeneity in the neurobiological mechanisms underlying both depression and AD [28]. Recent studies have begun to explore the relationship between cerebral microvascular changes and cognitive dysfunction in patients with MD, which may explain, at least in part, the elevated risk of AD observed in these individuals [31]. Cerebral microvascular dysfunction, often associated with cardiovascular risk factors such as hypertension and diabetes, could play a mediating role in this relationship [32,33]. This is particularly relevant in our sample, where a significant proportion of participants with depression also had cardiovascular comorbidities.

Several studies underscore the relationship between depression and the impairment of instrumental activities of daily living (IADLs) in older adults with dementia, especially regarding financial capacity, which is critical for maintaining independence. Financial decision-making, a complex IADL, declines significantly in individuals experiencing both depression and cognitive impairment, thereby increasing their vulnerability to exploitation and adverse health outcomes [3,34]. Addressing these functional declines early in patients with depression could be key to mitigating the onset of Alzheimer’s disease.

Compared to other longitudinal studies, our results are consistent with the findings of Sáiz-Vázquez et al. [10] who also found that major depression is associated with an increased risk of developing dementia. However, our research provides novel evidence that major depression may be associated with an even higher risk of developing AD, suggesting the importance of identifying subtypes of depression in clinical and epidemiological studies. We also note that the effects of depression on AD appear to be mediated, in part, by cardiovascular factors. This is consistent with the emerging hypothesis that cerebrovascular disease and depression may share common etiopathogenic pathways, such as microvascular changes and neuroinflammation [35].

Importantly, our analysis controlled for several well-known key confounders, including age, sex, and educational attainment. These factors are well established as modifiers of dementia risk, and their inclusion in multivariate models reinforces the validity of our findings [12]. In addition, we added cardiovascular and cognitive risk factors (such as MMSE score) to better understand how these elements inter-relate with depression in the onset of AD. Previous studies highlight the role of stress as a significant trigger of both depression and cognitive decline in older adults. Persistent systemic inflammation, which fuels ongoing neuroinflammation, is proposed as a key mechanism in this association [36]. This suggests that stress-related inflammatory processes may have influenced the outcomes in our cohort and should be carefully considered in future studies for their potential impact on neurodegenerative and depressive pathologies.

When interpreting the results, it is essential to consider certain limitations, as well as other relevant factors. First, the relatively low number of incident cases in some sociodemographic variables (e.g., unemployed or disabled individuals, or those from higher social classes) reduced the statistical power, although it did not prevent the detection of significant differences in the Cox regression models. The rejection rate, approximately 20%, from sampling to the start of Wave I is also substantial. However, this rejection rate was accounted for in the study design, and the losses and dropouts were comparable to similar studies [16,17]. In addition, while our diagnoses of depression and AD were based on validated tools, future research should consider more comprehensive diagnostic confirmation, including AD biomarkers and neuroimaging assessments. Lastly, the initial occupational data collected did not align with the categories used in some standardized instruments, so these data should be interpreted within the context of the analyses performed.

In terms of clinical implications, our findings highlight the importance of early assessment and treatment of depression in older adults, particularly those with psychotic symptoms. Health professionals should be aware of the possible link between major depression and the development of AD, and preventive interventions in these patients may be necessary to mitigate long-term risk. Previous studies suggest that early treatment of depressive disorders can reduce the risk of cognitive impairment [37], highlighting the relevance of integrating depression management into AD prevention strategies.

## 5. Conclusions

In conclusion, our results provide evidence for the relationship between major depression and an elevated risk of developing AD. These findings not only enrich the understanding of depression as a risk factor for dementia but also suggest that early interventions targeting specific subgroups of patients with depression could have a clinically significant impact on the prevention of cognitive decline. Additionally, future research should explore the mechanisms underlying this relationship, particularly the role of vascular and neurobiological pathways, to better understand how depression may contribute to neurodegenerative processes. This knowledge could inform the development of targeted preventive strategies and therapeutic interventions aimed at improving cognitive health and reducing the incidence of Alzheimer’s disease in high-risk individuals.

## Figures and Tables

**Table 1:** Participants’ basal sociodemographic and clinical characteristics.

		MD	Non-Cases	Sig.
Sig. Age, mean (s.d.)		71(8)	72(9)	
Sex, *n* (%)	Male	18(23.4)	1629(49.3)	**
	Female	59(76.6)	1673(50.7)	
Educational level, n (%)	Illiterate/some primary education	4(5.3)	245(7.5)	*
	Primary education	64(85.3)	2424(74)	
	Secondary education or higher	7(9.3)	605(18.5)	
Diabetes, *n* (%)		12(15.8)	383(11.7)	
Hypertension, *n* (%)		55(71.4)	2225(67.5)	
Previous heart disease, *n* (%)		7(9.7)	215(6.6)	
Previous vascular disease, *n* (%)		12(17.1)	347(10.9)	
Number of cardiovascular illness, *n* (%)	None	16(23.5)	924(29.2)	**
	1	42(61.8)	1913(60.6)	
	2	1(1.5)	35(1.1)	
	3	4(5.9)	146(4.6)	
	4	4(5.9)	106(3.4)	
	5	0(0)	33(1)	
	6	1(1.5)	2(0.1)	
MMSE score, mean (s.d.)		5(6.5)	72(2.2)	**

MD: major depression; s.d.: standard deviation; MMSE: Mini-Mental State Examination. *: *p* < 0.05; **: *p* < 0.001.

**Table 2:** Incident Alzheimer’s disease (AD) prediction according to major depression (MD) participants.

MD Status at Baseline	Model 1 ^a^		Model 2 ^b^		Model 3 ^c^	
	HR	95% CI	HR	95% CI	HR	95% CI
Non-case (*n* = 3302)	1	-	1	-	1	-
Case (*n* = 77)	2.91	1.03–8.24	3.21	1.13–9.12	2.81	0.98–8.01

HR: hazard ratio; CI (95%): confidence interval (95%); MD: major depression; ^a^ Model 1: include MD and established risk factors (age, gender, and educational level). ^b^ Model 2: model 1 and cardiovascular risk factors. ^c^ Model 3: model 2, sociodemographic risk factors, and MMSE score.

## Data Availability

The original contributions presented in the study are included in the article, further inquiries can be directed to the corresponding author.
